# Identification and Genome Phylogenetic Analysis of Three *Brucella abortus* Strains From Sheep, Yak, and Cow in Qinghai, China

**DOI:** 10.1155/tbed/7831968

**Published:** 2026-06-05

**Authors:** Xiaoan Cao, Qiuju Yang, Hongmei Xue, Lizhi Xu, Zhijie Liu, Jijun He, Youjun Shang, Zhenjun Li, Zhiguo Liu

**Affiliations:** ^1^ Veterinary Clinical Service Center, State Key Laboratory of Animal Disease Control and Prevention, Lanzhou Veterinary Research Institute, Chinese Academy of Agricultural Sciences, Lanzhou, 730046, China, caas.cn; ^2^ Department of Zoonosis Control and Prevention, Yunnan Provincial Key Laboratory for Natural Focal Disease Control and Prevention, Yunnan Institute of Endemic Disease Control and Prevention, Kunming, 650500, China; ^3^ Department of Brucellosis Control and Prevention, Qinghai Institute for Endemic Disease Prevention and Control, Xining, 810000, Qinghai, China, qhu.edu.cn; ^4^ Department of Bioinformatics, Beijing Macro and Micro-Test Bio-Tech, Beijing, 101599, China; ^5^ Branch of Biosafety, National Key Laboratory of Intelligent Tracking and Forecasting for Infectious Diseases, National Institute for Communicable Disease Control and Prevention, Chinese Center for Disease Control and Prevention, Beijing, 102206, China, chinacdc.cn

**Keywords:** average nucleotide value, *Brucella abortus*, multilocus sequence typing, phylogenetic analysis, whole genome sequence

## Abstract

Although brucellosis is endemic to the Qinghai–Tibet Plateau, the molecular epidemiology of circulating *Brucella abortus* is still poorly characterized. Thus, in this study, we adopted an integrated approach of bacteriology and whole‐genome sequencing (WGS) to genetically characterize *B. abortus* isolates from sheep, yak, and cattle in Qinghai, China. Conventional biotyping assays and multilocus sequence typing (MLST) show that the three strains were conclusively identified as *B. abortus* biovar 1, sequence type 2 (ST2). Furthermore, average nucleotide identity (ANI) analysis indicated that the three strains (BA0611, BAHYS, and BAQHM) showed ANI values above 99.99% and over 99.91% identity with the *B. abortus* reference strain, confirming their species assignment. The three *B. abortus* isolates BA0611, BAHYS, and BAQHM exhibited identical virulence gene profiles, harboring 69 virulence‐related genes altogether and uniformly lacking three crucial virulence‐associated genes, namely *bmaA*, *btpB*, and *virB10*. Core‐genome SNP phylogenetic analysis revealed close genetic relatedness to Tibetan strain XZ19‐1 and Russian isolates, distinguishing them from previously identified local human and marmot strains. The high genetic similarity observed among the three strains indicates a common source of infection, supporting the classification of these cases as a cluster infection. These data together confirm the prevalence of genetically divergent *B. abortus* lineages within the Qinghai–Tibet Plateau, which enriches current insights into the host spectrum and genomic diversity of this zoonotic pathogen in the region. These findings provide robust support for optimizing local genomic surveillance and formulating precise prevention and control strategies to reduce brucellosis prevalence in both animal and human populations.

## 1. Introduction

Brucellosis is a global zoonotic disease caused by bacteria of the genus *Brucella*, and it presents a major threat to public health and livestock economies across the world [1]. Globally, its annual incidence is estimated at ~1.6–2.1 million cases [2]. The disease is highly endemic across extensive regions of Asia and Africa, with particularly high incidence rates reported in Syria, Kyrgyzstan, Mongolia, Iran, Algeria, and Kenya [3]. Currently, Kuwait is also recognized as an endemic region where brucellosis continues to represent a persistent public health concern [4]. The sustained endemicity in these regions arises from a combination of factors, including high‐risk behaviors such as raw milk consumption, suboptimal livestock husbandry practices, including extensive farming and unhygienic slaughter, as well as broader systemic challenges such as constrained public health resources and high rates of misdiagnosis [5].

In China, following the adoption of effective control, human and animal brucellosis again emerged in the 1990s [6]. The geographical distribution of brucellosis endemicity in China has moved from pastoral regions (e.g., Inner Mongolia and Qinghai) to northern grassland and agricultural provinces (e.g., Hebei, Shanxi, and Liaoning) and, eventually, to southern provinces [7]. Qinghai, a vast pastoral and semipastoral region in China, is considered a high‐prevalence area for brucellosis, which is closely linked to large‐scale livestock farming and poor public hygienic conditions [8]. The incidence of human brucellosis sharply rose from 2.45 cases per 100,000 population in 2019–34.86 per 100,000 in 2023 [9]. An investigation further revealed the presence of at least three *Brucella* species (*Brucella melitensis*, *Brucella abortus*, and *Brucella suis*) in Qinghai, with *B. melitensis* considered the predominant species in the sampled area [8]. Phylogenetic analysis of 54 *B. melitensis* strains from Qinghai identified six subclades, including four distinct local lineages, indicating that the recent human brucellosis uptick has been largely driven by endemic historical transmission in these regions [10]. While *B. melitensis* is currently the main reported species, *B. abortus* still circulates in animal reservoirs and persists as a major zoonotic threat. A previous meta‐analysis estimated that the overall pooled seroprevalence of bovine brucellosis in China was 1.5% (95% CI: 0.6%–2.6%). Furthermore, a subgroup analysis showed variations in livestock type, with the highest seroprevalence being found in dairy cattle (3.1%), followed by yaks (1.5%) and beef cattle (1.3%) [11]. Additionally, a 2010 serosurvey on the Qinghai–Tibet Plateau of China, testing 621 yak serum samples from six counties using the serum agglutination test, demonstrated a 9% (56/621) seroprevalence of *Brucella* infection, indicating the commonality of brucellosis in the local yak population [12]. We previously isolated *B. abortus* from Himalayan marmots and human patients in the region and observed a 7.0% (80/1146) seroprevalence in marmots, along with molecular evidence pointing to frequent direct or indirect contact between domestic livestock (sheep, goats, and cattle) and marmots, suggesting that wildlife contributes markedly to the persistence and spread of *B. abortus* on the Qinghai–Tibet Plateau [13]. Even so, the lack of comprehensive genomic analysis of *B. abortus* strains isolated from multiple major domestic animal hosts limits our understanding of transmission dynamics and the exact role of wildlife in this multihost system. Whole‐genome sequencing (WGS) is now the preferred method for high‐resolution phylogenetic analysis and strain discrimination in the genetically monomorphic *Brucella* genus [14]. The current study establishes a comprehensive One Health genomic dataset, incorporating *B. abortus* isolates from human, domestic animal (i.e., sheep, yak, and cattle), and wildlife (marmot) hosts within the Qinghai–Tibet Plateau. To investigate transmission ecology in this multihost system, we adopted an integrated genomic approach. The initial characterization was done using multilocus sequence typing (MLST), followed by high‐resolution WGS and comparative pangenome analysis. This approach facilitates precise phylogenetic reconstruction and identification of the genomic variations associated with host adaptation and regional persistence, thus providing a powerful framework for clarifying pathogen epidemiology in complex ecological settings.

## 2. Materials and Methods

### 2.1. Specimen Collection and Bacterial Isolation

Splenic tissue samples from aborted sheep (*n* = 11), yak (*n* = 19), and cattle (*n* = 10) fetuses in Qinghai, China, in 2015–2016, were shipped on dry ice to the Lanzhou Veterinary Research Institute laboratory. Bacterial isolation followed standard brucellosis laboratory protocols [15]. Specifically, ~200 mg of spleen tissue was aseptically homogenized and resuspended in 500 µL of sterile phosphate‐buffered saline (PBS, 0.01 M, pH 7.2). Each homogenate was inoculated onto a *Brucella*‐selective medium (Oxoid, UK) and supplemented with 5%–10% horse serum. All procedures were implemented in a biosafety cabinet under strict aseptic conditions. Plates were incubated at 37°C under two atmospheric conditions: with 5%–10% CO_2_ and without CO_2_ supplementation. They were evaluated every 48 h for the appearance of translucent colonies for up to 14 days. Samples exhibiting no growth after this phase were deemed negative and sterilized through autoclaving before disposal.

### 2.2. Molecular Screening and Phenotypic Identification

Presumptive colonies were first classified as *Brucella* spp. using a genus‐specific BSCP‐31 PCR assay, producing a 223‐bp sized product [16]. Species‐level differentiation was then performed via the AMOS‐PCR method [17], which produces unique amplicons for specific *Brucella* species: 498 bp for *B. abortus* biovars 1, 2, and 4; 731 bp for the three biovars of *B. melitensis*; 285 bp for *B. suis* biovar 1; and 976 bp for *B. ovis*. The isolates then were put through conventional biotyping per conventional protocols, enabling us to evaluate the carbon dioxide requirement, hydrogen sulfide production, growth on media containing basic fuchsin or thionine dyes, agglutination with anti‐A and anti‐M monospecific sera, and vulnerability to *Brucella*‐specific phages (Tbilisi [Tb], Weybridge [Wb], *R*, and BK^2^) [18]. After phenotypic characterization and identification as *B. abortus*, pure bacterial cultures were inactivated by heating at 85°C for 10 min. Then, genomic DNA was extracted from the inactivated material using the QIAamp DNA Mini Kit (Qiagen, Germany) based on the manufacturer’s protocol. Furthermore, an adequate quantity of the purified DNA obtained was used as the template for WGS.

### 2.3. Genome Sequencing, Assembly, and Comparative Genomics

A sequencing library was assembled with the Nextera XT Library Preparation Kit (Illumina, USA) and underwent paired‐end sequencing (2 × 150 bp) on an MGISEQ‐2000 platform. Raw sequencing reads were assessed for quality using FastQC [19] (v0.11.9), and adapters and low‐quality bases were removed with Trimmomatic [20] (v0.39). De novo genome assembly was performed using SPAdes [21] (v3.15.5). The resulting genome was annotated with the Prokka pipeline [22] (v1.14.6). Average nucleotide identity (ANI) values were quantified against six reference *Brucella* genomes (*B. melitensis*, *B. abortus*, *B. suis*, *B. anis*, *B. ovis*, and *B. neotomae*), employing the Orthologous ANI Tool [23]. Antimicrobial resistance and virulence‐associated genes were determined through alignment against the Comprehensive Antibiotic Resistance Database [24] and virulence factor database (http://www.mgc.ac.cn/VFs/) [25] using thresholds of >98% coverage and sequence similarity. The core genes conserved among all isolates in the analysis were identified using Panaroo [26] (v1.2.10).

### 2.4. Molecular Genotyping and Phylogenetic Inference

*In silico* MLST was conducted on the assembled genome based on the standard scheme (https://pubmlst.org/brucella/) [27]. Furthermore, a total of 550 *B. abortus* genome sequences, including the isolate from this study, were derived from the NCBI GenBank database (Table S1). For phylogenetic analysis, *B. abortus* 544 (GCA_000369945.1) was adopted as the reference genome to identify core genome single nucleotide polymorphisms (cgSNPs) with Snippy v4.6.0. Recombination‐linked SNPs were identified and discarded by Gubbins v2.0.0 [28], and a recombination‐free SNP alignment was subsequently produced through snp‐dists v0.8.2. A maximum‐likelihood phylogenetic tree was inferred from this recombination‐free SNP matrix using IQ‐TREE v2.2.0 [29] with 1000 bootstrap replicates, and the resultant tree was visualized via iTOL v6.5.7 [30].

## 3. Results

### 3.1. Species Identification and ANI Analysis of *B. abortus* Strains

**Figure 1:**
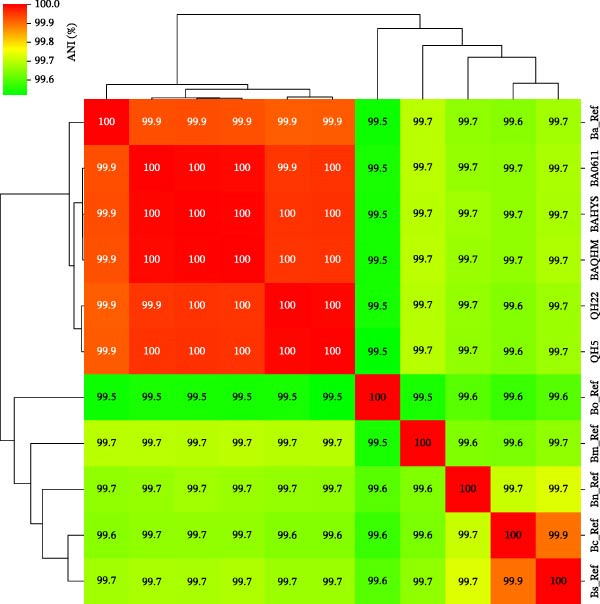
Comparative analysis of average nucleotide identity (ANI) analysis of *B. abortus* strains. Note: Figure showing the ANI of strains in this study to other five classical *Brucella* species, including (*B. melitensis* [Bm], *B. suis* [Bs], *B. canis* [Bc], *B. ovis* [Bo], and *B. neotomae* [Bn]). The numerical labels in the figure represent the ANI values calculated from whole‐genome comparisons between each pair of strains.

BSCP‐31 PCR produced a 223 bp product, thus confirming the isolates as *Brucella* spp., while AMOS‐PCR amplified a specific 498 bp fragment, matching the profile of the reference strain *B. abortus* biovar 1. Based on phenotypic characteristics, the isolate was identified as *B. abortus* biovar 1 (Table 1). Based on the ANI analysis, the results showed a high degree of genomic similarity among the examined strains and reference strains of *Brucella*. The three study strains (BA0611, BAHYS, and BAQHM) demonstrated ANI values exceeding 99.99%, thus verifying their close genetic relationship. Moreover, they exhibited high ANI values (> 99.91%) with the *B. abortus* reference strain (Ba_Ref; Figure 1), thus supporting their classification within this species. However, ANI values with reference strains of other *Brucella* species, such as *B. canis* (Bc_Ref), *B. melitensis* (Bm_Ref), *B. neotomae* (Bn_Ref), *B. ovis* (Bo_Ref), and *B. suis* (Bs_Ref)—were consistently lower, at ~99.54%–99.89%. The strains QH22 and QH5 also showed extremely high ANI values (>99.99%) with each other and high similarity (>99.91%) with the *B. abortus* reference and the three main study strains.

**Table 1:** Phenotypic characteristics of the three *B. abortus* strains in the present study.

Key	CO_2_	H_2_S	Growth in the presence of dyes		Monospecific sera			Lysis by phage at RTD			AMOS‐PCR	Number of strains	Conclusion
			Fuchsin 20 µg/ml	Thionin 20 µg/ml	A	M	R	Tb	BK_2_	Wb			
*B. abortus* 544	+	+	+	−	+	−	−	CL	CL	CL	489 bp	1	*B. abortus* bv. 1
*B. melitensis*16 M	−	−	+	+	−	+	−	NL	CL	NL	731 bp	1	*B. melitensis* bv. 1
*B. suis* 1330	−	+	−	+	+	−	−	NL	CL	CL	258 bp	1	*B. suis* bv. 1
BA0611	+	+	+	−	+	−	−	CL	CL	CL	498 bp	1	*B. abortus* bv.1
BAHYS	+	+	+	−	+	−	−	CL	CL	CL	489 bp	1	
BAQHM	+	+	+	−	+	−	−	CL	CL	CL	489 bp	1	

*Note*: CO_2_ requirement, H_2_S production; “+”: positive; “−”: negative; agglutination with monospecific A, M, and R (rough) antisera.

Abbreviations: CL, complete lysis; NL, not lysis; RTD, routine test dilution.

### 3.2. Genome Features and Pan‐Genomic Analysis of *B. abortus* Strains

The genome assembly characteristics of the *B. abortus* strains showed high consistency and quality. The assemblies were made up of 18–22 scaffolds, with N50 values ranging from 297,696 to 383,549 bp and N75 values ranging from 217,106 to 253,391 bp. The largest scaffold measured between 391,683 and 631,600 bp, while the total genome size remained stable at ~3.26–3.27 Mb. The GC content varied slightly from 55.3% to 56.1%, while the gap frequency was 0–30 N/100 kb, reflecting the well‐organized and highly contiguous genomic assemblies typical of *Brucella* species.

**Figure 2:**
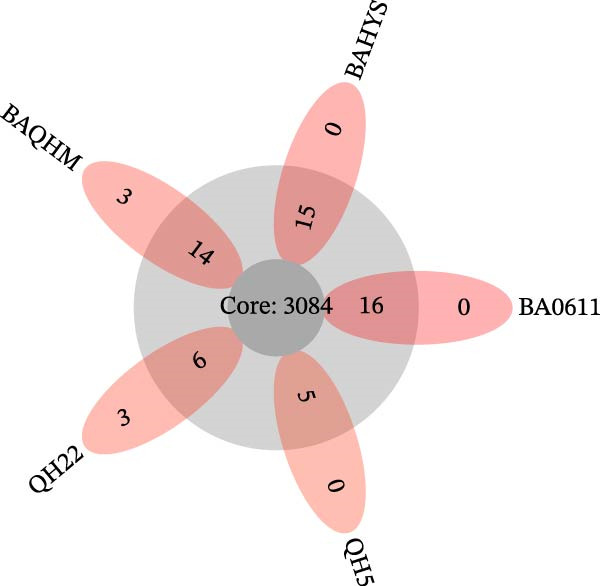
Shows the results of a pan‐genomic analysis conducted on five *B. abortus* strains from Qinghai. The Venn diagram visualizes the distribution of core and unique genes, with the shared core set positioned centrally and strain‐specific genes quantified in the peripheral segments.

The pan‐genomic analysis showed a highly conserved genetic structure among the analyzed *Brucella* strains. The total pan‐genome comprised 3103 genes, of which 3084 made up the core genome (present in 99% to 100% of strains), while no soft‐core genes (95% to <99%) were observed (Figure 2). The accessory genome was limited to 23 shell genes (present in 15% to <95% of strains), with no cloud genes (0% to <15%) identified. Moreover, strain‐specific analysis demonstrated variations in accessory gene content across the studied strains. BAQHM carried 14 accessory genes, along with BAHYS 15 and BA0611 16, with three unique genes identified only in BAQHM.

### 3.3. Predicted Virulence‐Associated Genes and Antimicrobial Resistance Genes of *B. abortus* Strains

**Figure 3:**
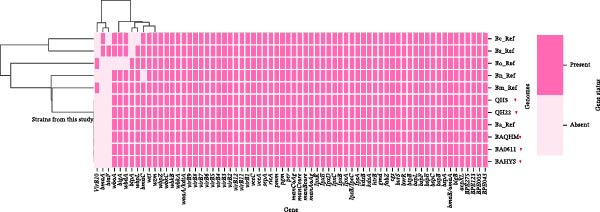
Shows the virulence‐associated gene repertoire of the Qinghai strains. The analysis identified 30 genes linked to lipopolysaccharide biosynthesis, 13 encoding T4SS secreted effectors, 11 constituting the VirB type IV secretion system, six involved in Brucebactin siderophore synthesis, and two comprising the *bvrR/bvrS* regulatory system. In addition, seven unique virulence factors were each represented by a single gene.

The virulence factor analysis showed that the three *B. abortus* strains (BA0611, BAHYS, and BAQHM) shared the same virulence gene profile and contained 69 virulence‐associated genes (Figure 3 and Table 2). Moreover, all strains consistently lacked three key virulence‐associated genes: *bmaA*, *btpB*, and *virB10*. Even so, the strains delivered a complete complement of other critical virulence genes, including those involved in lipopolysaccharide biosynthesis (i.e., *lpx* cluster, man cluster, and *wbk* cluster), the remaining components of the type IV secretion system (i.e., *virB* operon), and regulatory functions (i.e., *bvrR/bvrS*). This conserved profile, characterized by the specific triple‐gene absence (*bmaA* ^−^, *btpB* ^−^, and *virB10* ^−^), is indicative of a highly clonal and genetically distinct lineage in the *B. abortus* population in this region.

**Table 2:** The composition profile of virulence‐associated genes of the three *B. abortus* in the present study.

Virulence factors	Gene counts	Virulence genes	Biology function
LPS (VF0367)	30	*lpsA*, *A*, *wbpZ*, *acpXL*, *wboA*, *wbpL*, *pmm*, *manAoAg*, *pgm*, *lpsB/lpcC*, *fabZ*, *per*, *wbdA*, *lpxD*, *lpxE*, *lpxK*, *manBcore*, *wzm*, *htrB*, *wzt*, *wbkC*, *wbkB*, *kdsA*, *wbkA*, *manCcore*, *kdsB*, *lpxB*, *lpxC*, *lpxA*, *gmd*	Immune modulation
T4SS secreted effectors (VF0695)	13	*ricA*, *bspL*, *bspJ*, *bspE*, *bspC*, *bspA*, *bspB*, *sepA*, *BPE005*, *BPE123*, *vceA*, *BPE275*, *BPE043*	Effector delivery system
VirB type IV secretion system (VF0365)	11	*virB1*, *virB2*, *virB3*, *virB4*, *virB5*, *virB6*, *virB7*, *virB8*, *virB9*, *virB11*, *virB12*	Effector delivery system
Brucebactin (VF0692)	6	*entD*, *dhbE*, *dhbC*, *dhbB*, *dhbA*, *vibH/entF*	Nutritional/metabolic factor
BvrR‐BvrS (VF0368)	2	*bvrR*, *bvrS*	Regulation
Direct heme uptake system (VF0693)	1	*BABS19_RS15905*	Nutritional/metabolic factor
BmaC (VF1341)	1	*bmaC*	Adherence
BigB (VF1345)	1	*bigB*	Adherence
CG (VF0366)	1	*cgs*	Immune modulation
BtpB (VF0522)	1	*btpB*	Immune modulation
SP41 (VF0694)	1	*ugpB*	Adherence
BigA (VF1344)	1	*bigA*	Adherence

Furthermore, the analysis of antimicrobial resistance genes in the sequenced *Brucella* isolates revealed a consistent finding across all strains (BA0611, BAHYS, and BAQHM). That is, all strains were found to carry the *mprF* gene, which is homologous to the *mprF* gene originally characterized in *B. suis*. This gene contributes to resistance against peptide antibiotics by fostering lysylphosphatidylglycerol synthesis and later modification of the bacterial membrane.

### 3.4. In Silico MLST and cgSNP Phylogenetic Analysis of *B. abortus* Strains

**Figure 4:**
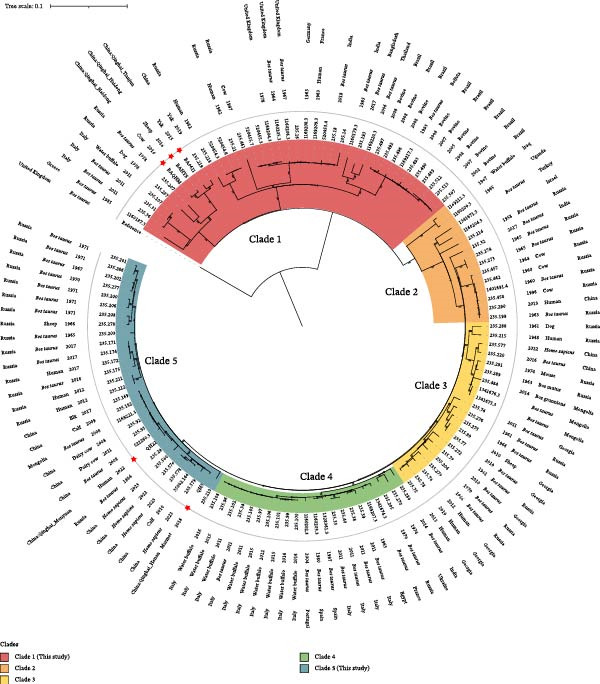
The cgSNP‐based maximum‐likelihood phylogeny constructed from *B. abortus* strains belonging to a specific clade, with branch support derived from 1000 bootstrap replicates. In the tree, strains sequenced in the present study are highlighted in red star and isolation hosts marked with bold black.

Based on an MLST analysis, all strains shared an identical allelic profile, corresponding to sequence type 2 (ST2), with the following allele combination: *gap* (2), *aroA* (1), *glk* (2), *dnaK* (2), *gyrB* (1), *trpE* (3), *cobQ* (1), *int_hyp* (1), and *omp25* (1). ST2 is known to be geographically widespread across Asia and Europe. Global cgSNP phylogenetic analysis showed that the five Qinghai strains clustered within a single clade, which was further classified into two distinct subclades (Figure S1 and Figure 4). Three of the animal‐derived strains exhibited close genetic relatedness to *B. abortus* strains isolated from yak in Tibet in 2019, as well as to strains from Russia (including 235.287 and 235.207; Figure 4). Moreover, strain QH5 was phylogenetically the closest to isolates from Ningxia and Heilongjiang, while QH22 was clustered with strains from Inner Mongolia, Hebei, and Russia. The identification of these strains evidences the co‐circulation of distinct *B. abortus* lineages on the Qinghai–Tibet Plateau.

## 4. Discussion

This study represents the first systematic isolation and characterization of *B. abortus* strains from three hosts in Qinghai, China. The identification of these strains expands the known host spectrum of *B. abortus* and also highlights previously unrecognized genetic diversity in this high‐altitude ecosystem. Brucellosis was found to impose a substantial economic burden on livestock. For example, in the study area, the estimated annual cost attributed to brucellosis in yaks was 521,043 USD, averaging 1.42 USD per yak [31]. Moreover, dairy cattle exhibited the highest brucellosis seroprevalence (3.1%), surpassing beef cattle (1.3%) and yaks (1.5%). Furthermore, regions with vaccination programs had higher rates of brucellosis (1.8%) than those without (0.5%). Moreover, a concurrent rise in both cattle and human cases was observed [32]. In our previous survey, serological testing showed a brucellosis seroprevalence of 7.0% (80/1146) in marmots in Qinghai, from which one *B. abortus* strain was isolated. An additional strain was obtained from a human patient [13]. At the same time, 577 human brucellosis cases were recorded in Qinghai over 2005–2019 (average prevalence: 0.07/100,000), with herdsmen being the most affected occupational group at 47.83% (276/577) [33]. From a public health perspective, these results highlight the need for enhanced surveillance in remote pastoral regions, where brucellosis presents a sustained risk to animal production and human health.

Pan‐genomic analysis showed a highly conserved genomic structure between the isolates, with a minimal accessory gene pool and no cloud genes detected, indicating genetic stability and clonality. Even so, strain‐specific variations in accessory gene content were found, which may contribute to high‐altitude adaptations. Notably, all strains shared an identical virulence gene profile characterized by the consistent absence of *bmaA*, *btpB*, and *virB10*, whose notable absence may reflect lineage‐specific adaptation to local hosts or environmental conditions [34]. For example, previous research has shown that the more severe colonization defect observed in a *virB10* mutant, relative to a *virB1* mutant, establishes *VirB10* as a critical and nonredundant component of the *B. abortus* T4SS, which is essential for establishing chronic infection [35]. Future work should thus assess if this holds true for strains endemic to the Qinghai–Tibet Plateau, where environmental pressures may have shaped distinct virulence strategies. The ubiquitous distribution of the *mprF* gene across isolates points to a conserved genetic determinant for intrinsic resistance to cationic peptides, which is likely mediated by bacterial membrane charge alteration. Moreover, a transcriptomic study of rifampicin shows a link between induced gene expression changes and major cellular functions, including core metabolism, catalysis, and membrane integrity [36]. These genomic features provide insights into the evolutionary mechanisms that allow *B. abortus* to persist in plateau ecosystems.

Furthermore, MLST analysis classified all isolates into ST2, a sequence type that has been documented widely across Asia and Europe, thereby supporting the hypothesis of the historical and ongoing spread of this lineage [37]. A previous study showed that nine *B. abortus* strains collected from Xinjiang (China) were recognized as ST2 [38]. These data suggest that surveillance efforts should prioritize ST2 *B. abortus* in the northwest region. Moreover, the cgSNP phylogeny further resolved the Qinghai strains into two well‐supported subclades with distinct phylogeographic and host patterns. The first subclade, comprising strains from sheep, yak, and cow, was genetically linked to strains from Tibet and Russia. The second, composed of human and marmot strains, was clustered with isolates from northern and eastern China. This substructure pointed to several independent introduction events into the plateau, followed by localized diversification rather than one recent emergence. The close genetic relationship witnessed between the Qinghai isolates and strains from Tibet and Russia indicated possible transregional transmission pathways, which may be facilitated by livestock movement and trade. Compared with earlier Chinese isolates, XZ19‐1 demonstrated closer genetic relatedness to *B. abortus* strains of European origin [39]. *B. abortus* isolated from Ningxia was more closely phylogenetically related to strains from other parts of Asia and Europe [40]. Moreover, genetic analyses showed that *B. abortus* strains from Kazakhstan and Russia cluster with isolates from Portugal, Brazil, and the United States. This pattern indicates a bidirectional ancient dispersal from Europe, with one lineage spreading westward to the Americas and another moving eastward to Asia via Russia [41]. The sustained cocirculation of these related but distinct sublineages reflects a dynamic and continually evolving population of *B. abortus* in the region, likely driven by factors such as cross‐regional livestock trade, seasonal grazing patterns, ecological adaptations to high‐altitude environments, and potential transmission interfaces with wildlife reservoirs. Owing to the recurring spillover of *B. abortus* from wildlife reservoirs into domestic cattle, the Greater Yellowstone Area is an ongoing obstacle to the complete elimination of bovine brucellosis in the United States [42]. These results highlight the importance of integrating genomic surveillance with movement and ecological data to better understand and manage the brucellosis spread in complex plateau ecosystems. The simultaneous presence of *B. abortus* in multiple hosts (i.e., livestock, wildlife, and humans) and environmental matrices within the Qinghai region creates a self‐sustaining regional transmission cycle. To mitigate the persistent threat of brucellosis, a transformative shift toward proactive, One Health‐based prevention is essential, involving the implementation of rigorous cross‐border biosecurity and a robust genomic surveillance system. This integrated strategy will aid in the transition from reactive containment to predictive prevention, thus reducing the notable human and economic burden of this neglected zoonosis.

Although this study provides important insights into the epidemiological characteristics of brucellosis in this region, several limitations should be noted. First, the relatively limited number of strains examined limits the generalizability of the findings. Second, the study lacks phenotypic antibiotic susceptibility data for the isolates, which are crucial for determining the effective antibiotics circulating in the region and providing valuable epidemiological guidance for treatment and control. Future research must expand both the geographic scope and sample size of surveillance to achieve a representative isolate collection, and it must include standardized antibiotic susceptibility testing.

## 5. Conclusion

The present study provides the first genomic characterization of *B. abortus* strains from livestock in the Qinghai–Tibet Plateau, revealing a clonal population with a substructure that is related to strains from Tibet, Russia, and other regions of China. These results enhance our understanding of *Brucella* diversity and transmission in high‐altitude ecosystems and offer a genetic basis for improving local control measures. The concurrent presence of *B. abortus* in diverse hosts and environmental reservoirs sustains a regional transmission cycle in Qinghai. Addressing this challenge must adopt a multipronged strategy expanding pathogen isolation across hosts and regions, analyzing strain distribution in conjunction with environmental drivers, and conducting functional studies to evaluate the impact of specific genetic deletions and resistance traits.

## Author Contributions

Xiaoan Cao and Hongmei Xue were responsible for strain isolation, biotyping, DNA preparation, and genome sequencing. Zhiguo Liu and Qiuju Yang drafted the initial manuscript and prepared the figures. Zhiguo Liu and Lizhi Xu charged the bioinformation analysis and visualizations. Zhenjun Li, Zhijie Liu, Jijun He, and Youjun Shang performed the epidemiological investigation. Zhiguo Liu, Zhenjun Li, and Xiaoan Cao designed the study and provided critical revisions.

## Funding

This work was financially supported by the National Key R & D program of China (Grant 2025YFD1800102), the National Natural Science Foundation of China (Grant U23A20237), the Central Guiding Local Science and Technology Development Program of Ningxia (Grant 2024FRD05072), the Earmarked Fund for the China Agriculture Research System (Grants CARS‐39‐13 and CARS‐39‐04), and the Gansu Provincial Science and Technology Special Mission (Grant 25CXNA006).

## Disclosure

The funding bodies were not involved in study design, data collection, analysis, interpretation, manuscript writing, or the decision to publish. All authors reviewed, edited, and approved the final version of the manuscript.

## Ethics Statement

This research was a retrospective molecular epidemiological investigation of historically archived strains, employing WGS and SNP analysis. All study procedures obtained prior approval from the Animal Ethics Committee of the Lanzhou Veterinary Research Institute, Chinese Academy of Agricultural Sciences (LVRIAEC‐2023‐036), and strictly complied with institutional guidelines and local regulatory standards.

## Conflicts of Interest

The authors declare no conflicts of interest.

## Supporting Information

Additional supporting information can be found online in the Supporting Information section.

## Supporting information


**Supporting Information 1** Table S1: Maximum‐likelihood phylogenetic analysis of 550 *B. abortus* strains was conducted in this study.

**Supporting Information 2** Figure S1: A global maximum‐likelihood phylogenetic tree of 550 *B. abortus* strains (Table S1) (constructed with IQ‐TREE), including strains from this study and global references. This panel depicts an expanded view of the clade (circled in the global tree) that contains all *B. abortus* strains isolated in this study, extracted for clearer visualization of their phylogenetic relationships.

## Data Availability

The WGS datasets generated and analyzed during this study are publicly available in the GenBank repository under the BioProject accession code PRJNA930323.
