# Supplementary figures and images for: Identification and Genome Phylogenetic Analysis of Three Brucella abortus Strains From Sheep, Yak, and Cow in Qinghai, China
Source: Transbound Emerg Dis. 2026 Jun 5;2026:7831968. doi: 10.1155/tbed/7831968 (PMC13238248; doi:10.1155/tbed/7831968)

Clades

- Clade 1
- Clade 2
- Clade 3
- Clade 4
- Clade 5

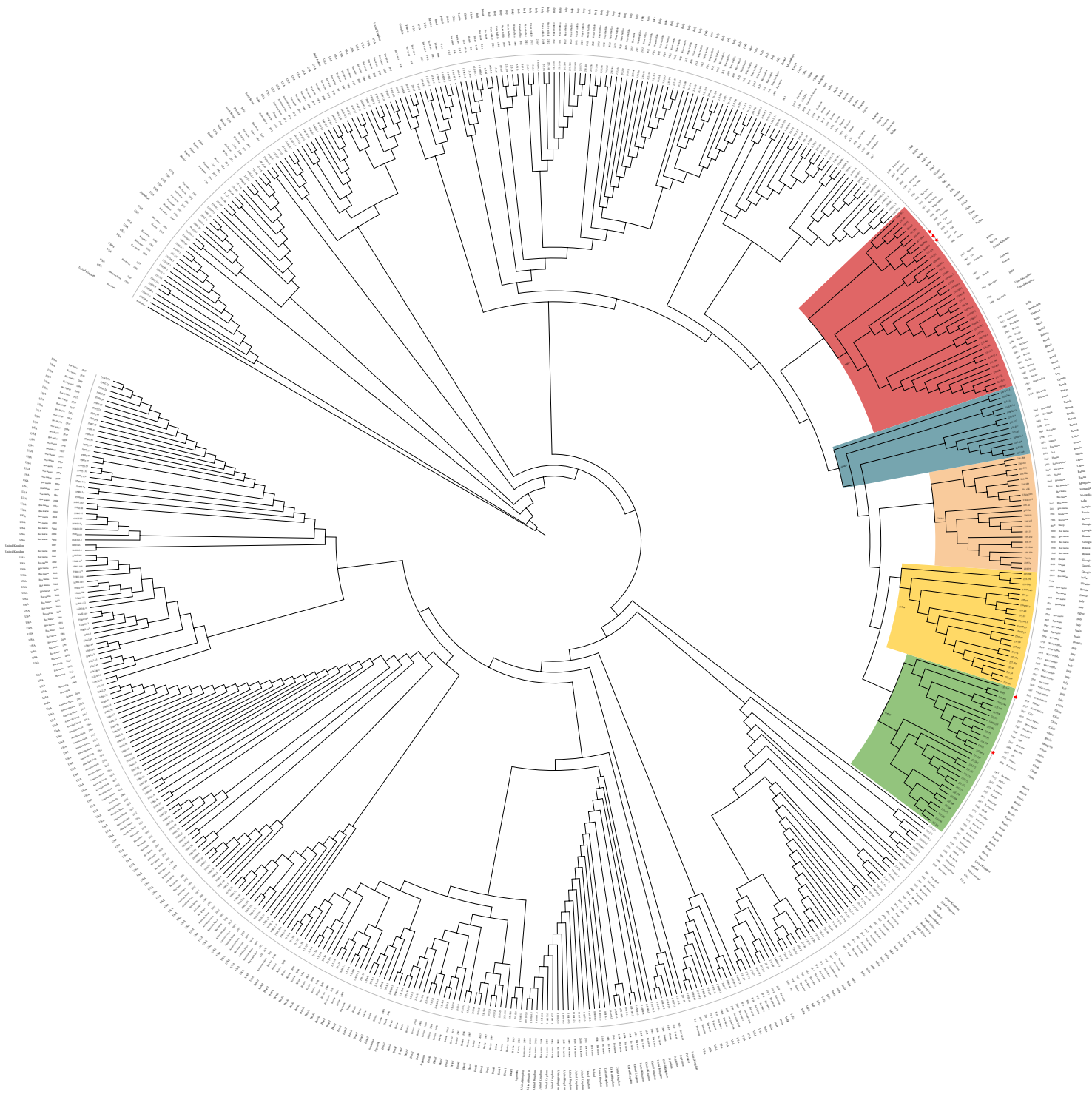

Supplement: Supplementary file 2 — Supporting Information 2 Figure S1: A global maximum‐likelihood phylogenetic tree of 550 B. abortus strains (Table S1) (constructed with IQ‐TREE), including strains from this study and global references. This panel depicts an expanded view of the clade (circled in the global tree) that contains all B. abortus strains isolated in this study, extracted for clearer visualization of their phylogenetic relationships. [file TBED-2026-7831968-s001.pdf]
